# Social Network: a Cytoscape app for visualizing co-authorship networks

**DOI:** 10.12688/f1000research.6804.3

**Published:** 2015-12-23

**Authors:** Victor Kofia, Ruth Isserlin, Alison M.J. Buchan, Gary D. Bader

**Affiliations:** 1 The Donnelly Centre, University of Toronto, Toronto, ON, M5S 1A8, Canada; 2 Faculty of Medicine, University of Toronto, Toronto, ON, M5S 1A8, Canada

**Keywords:** social network analysis, data visualization, collaborative networks, co-publication networks

## Abstract

Networks that represent connections between individuals can be valuable analytic tools. The Social Network Cytoscape app is capable of creating a visual summary of connected individuals automatically. It does this by representing relationships as networks where each node denotes an individual and an edge linking two individuals represents a connection. The app focuses on creating visual summaries of individuals connected by co-authorship links in academia, created from bibliographic databases like PubMed, Scopus and InCites. The resulting co-authorship networks can be visualized and analyzed to better understand collaborative research networks or to communicate the extent of collaboration and publication productivity among a group of researchers, like in a grant application or departmental review report. It can also be useful as a research tool to identify important research topics, researchers and papers in a subject area.

## Introduction

A scientist’s research output and collaborative tendencies - at least those that can be measured based on publications - can be visually summarized as a network where each node denotes an author and edges link authors who have co-published. Such a network facilitates determining who publishes with whom and in what topics, and identifying key individuals and organizations within collaborative research networks. It is useful to create and visualize a network showing a broad overview of collaborative research publications to communicate the extent of collaboration and impact of publications. As another example, creating a collaboration network from a set of publications for a specific topic, for example “Alzheimer’s”, can help highlight experts in the field and could be useful as a research tool to help identify important topics, researchers and papers.

Previously, creating co-authorship networks required users to manually retrieve the relevant data and transform it into either a formatted text file or an excel workbook that defined all the individual nodes and connections. Users would then have to import the text file or workbook into Cytoscape or another network visualization tool. To streamline this workflow, we developed the Social Network app, a Cytoscape 3 app that is capable of automatically generating visual summaries of individuals connected in academia. In the simplest mode of interaction, the user supplies the first initial and last name of the individual whose network they would like to visualize, and a co-authorship network is generated automatically from one of three currently supported bibliographic databases: PubMed, Scopus and Web of Science (via InCites). Users can also provide more complex and larger sets of publications, for example using the PubMed query system.

## Methods and implementation

### User interface

**Figure 1.**
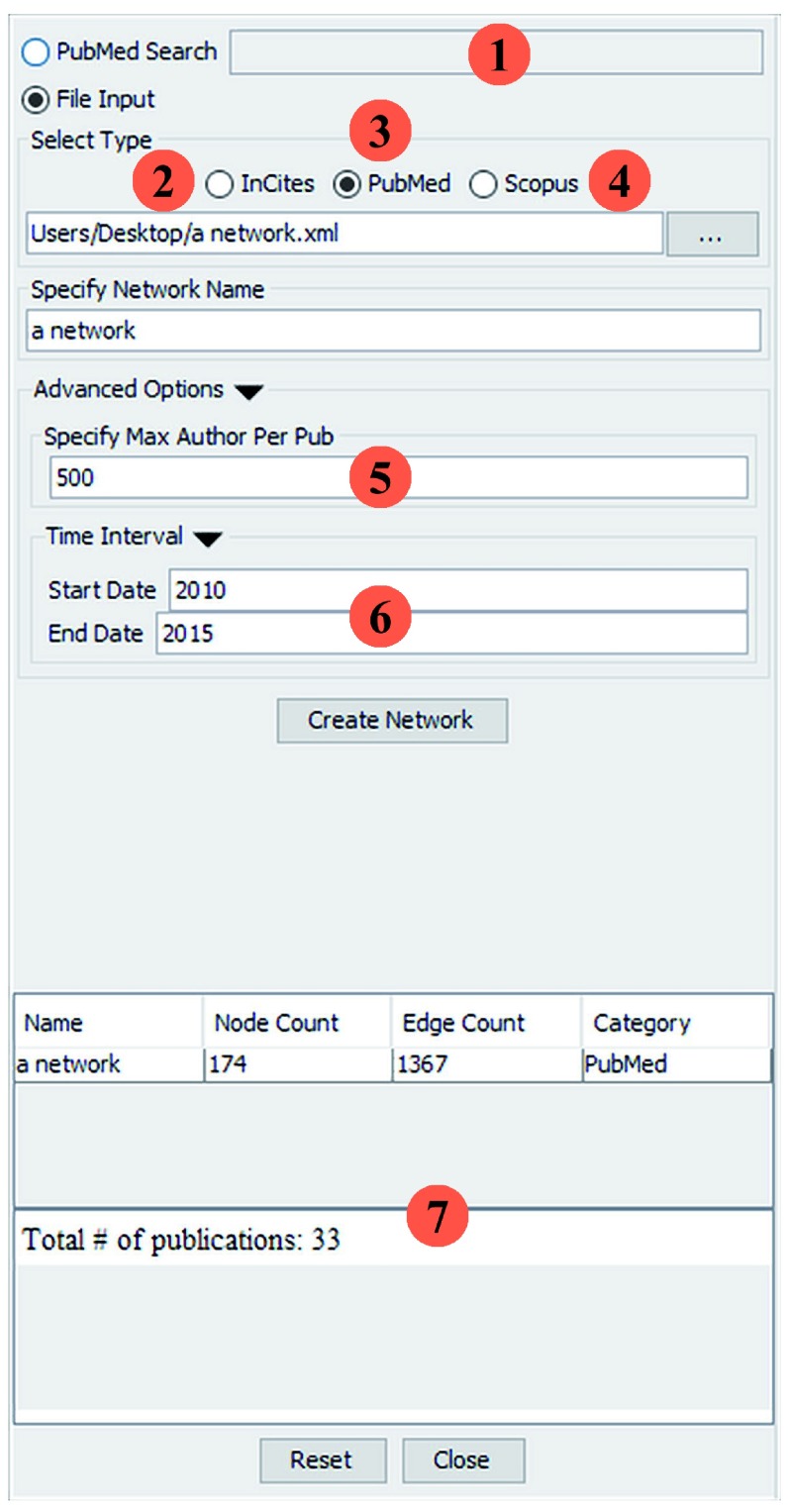
Snapshot of the Social Network user interface. Co-authorship networks can be generated in four ways: (1) By entering a query into the PubMed search box. (2) Loading an InCites report (XLSX format). (3) Loading a PubMed XML file containing query results retrieved from the PubMed web interface. (4) Loading a Scopus CSV file. (5) Users can also set the maximum # of authors allowed for a publication to filter out very large author lists that may clutter the network. (6) Users can specify a time interval for the co-authorships, which can optionally be visualized as bar charts on each author node showing number of publications plotted against publication year. (7) Extra information associated with each network is displayed in the network summary panel.

The Social Network App supports both text and file-based inputs. As Figure 1(1–4) shows, co-authorship networks can be created in four ways. With the search box, users can run queries against PubMed. A co-authorship network is automatically generated from any results that are retrieved. Alternatively, users can go directly to PubMed, Scopus or InCites web sites, search for publications, export them to a specified file format and visualize them using the app. See the user guide for detailed instructions on how to do each of these tasks ( http://baderlab.org/UserguideSocialNetworkApp);

Often when running queries with common names (e.g. “Smith J”), the query returns publications that contain more than 500 authors. Visualizing networks containing these types of publications is challenging because generating *n* · ( *n* - 1)/2 edges for each publication ( *n* refers to the # of authors in the publication) is resource intensive and the large clusters created are difficult to visualize and interpret. To avoid this issue, the user panel includes a maximum author per publication field that allows users to specify the author number threshold at which publications are excluded. By default the threshold is set to 500.

A co-authorship network summary panel is also included in the user panel ( Figure 1(7)). For PubMed and Scopus networks, the panel displays the total number of publications parsed and the number of excluded publications (publications are excluded if the number of authors they have exceeds the threshold). For direct PubMed queries the panel also includes the query translation automatically performed by PubMed. Scopus and InCites networks contain institutional affiliations for all the authors of a given publication. For InCites networks, charts that summarize the total number of publications and citations by location can be viewed by clicking on links in the panel that navigate to summary charts created with the Google Chart API ( https://developers.google.com/chart/).

### Implementation

Social Network is written in the Java programming language as an app for Cytoscape 3 ^1^ and is based on the Cytoscape 3-supported OSGi (Open Services Gateway Initiative) software architecture. To facilitate development, we developed a set of coding guidelines and defined them as Eclipse templates. The Eclipse templates and instructions on how to import them into an existing workspace are available at ( http://baderlab.org/Software/SocialNetworkApp/Development). We also used the Maven project management tool ( https://maven.apache.org/) to retrieve and organize the dependencies required by the app. An outline of the required dependencies is provided in a pom file that is located in the project source code ( https://github.com/BaderLab/SocialNetworkApp).

The design of the app followed Object Oriented Principles (OOP), reflected in the following class hierarchy; To facilitate future support for other social network types, we defined a set of flexible data structures, namely: AbstractNode, AbstractEdge and SocialNetwork. These data structures are used to represent networks and conveniently associated data created by the app in a general form. They are also specialized for each network type (e.g. PubMed, InCites). We also implemented the PubMed search feature generally to support future social network sources. Implementation details are provided in the source code ( https://github.com/BaderLab/SocialNetworkApp).

Networks built from different bibliographic databases require their own unique visual styles. We implemented a standard visual style that all other visual styles extend. The standard visual style describes styles for attributes that all social networks share (e.g. *name, label*) and it is used for PubMed and Scopus networks but not InCites networks. InCites networks require their own specialized visual style because they contain additional attributes ( *location*) that PubMed and Scopus networks typically do not possess. We implemented a new InCites visual style by extending the standard visual style and adding new style descriptions for the locations of authors. See the source code for implementation details.

### Database evaluation and implementation

Multiple bibliographic databases were evaluated for support by the app: PubMed, Scopus, Web of Science/InCites, and Google Scholar. Evaluation was based on application program interface (API) availability, data export capabilities, coverage, citations and update frequency (see Table 1). PubMed is developed and maintained by the National Center for Biotechnology Information (NCBI) as part of the U.S. National Library of Medicine and is accessible through the Entrez query system ( http://www.ncbi.nlm.nih.gov/pubmed/). Web of Science is a literature citation index created by Thomson Reuters containing over 90,000,000 records from all fields of science ( http://wokinfo.com/citationconnection/realfacts/). Web of Science data is also accessible via the Thomson Reuters InCites web-based search engine, which facilitates access to additional information, such as author institution ( http://researchanalytics.thomsonreuters.com/incites/). Scopus contains over 57,000,000 records, 27 million of which are patent records and 6.8 million of which are conference papers or proceedings ( http://www.elsevier.com/solutions/scopus/content) and date back as far as 1823 ( http://www.elsevier.com/__data/assets/pdf_file/0007/69451/sc_content-coverage-guide_july-2014.pdf).

**Table 1.** Coverage of various bibliographic databases. Scopus temporal coverage was retrieved here http://www.elsevier.com/__data/assets/pdf_file/0007/69451/sc_content-coverage-guide_july-2014.pdf.

Database	Indexed Citations	Temporal Coverage
**PubMed**	over 24 million	1946-present
**Web of Science**	over 90 million	1900-present
**Scopus**	over 57 million	1823-present
**Google Scholar**	100 million–160 million ^2, 3^	1700-present ^3^

Scopus contains author profiles that include, among other things, the institutional affiliations of an author. These profiles are helpful when disambiguating authors with very similar or identical names. Web of Science, InCites and Scopus access requires a paid subscription, which large academic institutions often provide. Google Scholar is a freely available and automatically updated database of citations with associated author pages ( https://scholar.google.com/intl/en/scholar/about.html).

Database content was evaluated by selecting a specific publication and comparing its citation counts among the different databases. Update frequency was determined by checking whether a newly published paper (published on January 1st 2015 or later) had been indexed by the database and by examining citation counts and verifying that newer citations had been captured. Prior to app development, data from PubMed, Web of Science and Scopus was available for an internal project. Thus, development was oriented towards supporting content from these three databases.

Aside from Google Scholar, every database we examined had an API. Developers can access the APIs provided by both Scopus and Web of Science but a subscription is required. On the other hand, PubMed content and API access is free, easing implementation. Although both Scopus and Web of Science require paid subscriptions to view their data over the web, often large institutions have licenses to query this data which makes it accessible to many users. Scopus and Web of Science also both provide an intuitive web-based user interface that enables users to export the data to file formats that are recognizable by our app (CSV for Scopus and XLSX for Web of Science). Based on our evaluation, we chose to support PubMed (via file export and API), Scopus and Web of Science (via file export). We would have supported Google Scholar if a public API or file export was available.

PubMed is the default search engine used by the app because of the accessibility of its content and the straightforward nature of its associated retrieval mechanisms: its web-based interface and the Entrez Programming Utilities (eUtils) API. Eutils enables URL-based (non-RESTful) programmatic access to data contained in PubMed as well as any other databases linked to Entrez ( http://www.ncbi.nlm.nih.gov/books/NBK1058/). Standard PubMed queries, for example “LastName First Initial”[Au], including recognized PubMed search tags ( http://www.nlm.nih.gov/bsd/mms/medlineelements.html), can be entered into the PubMed search field in the app, which retrieves XML results using the eUtils web service. The results are parsed using the SAX (Simple API for XML) API included in the Java standard library and are transformed into a co-authorship network using the Cytoscape API. Nodes in the network represent authors, edges represent co-authorship and how frequently authors collaborate is indicated by the thickness of an edge.

Because data is retrieved from the NCBI servers through POST calls there is no restriction on the length of queries passed to PubMed through the app ( http://www.ncbi.nlm.nih.gov/books/NBK25499/). Users can also construct networks from XML files exported directly from PubMed. Instructions for this workflow are provided in the app user guide: http://baderlab.org/UserguideSocialNetworkApp#PubMed. XML results obtained through eUtils differ slightly from XML results directly exported from PubMed. In particular, XML results exported from PubMed do not contain citations, whereas XML results retrieved by eUtils do. To correct this, the app retrieves this information using eUtils. Since the citation counts ultimately come from the same source regardless of how the initial data was obtained (PubMed or eUtils), networks generated via either method are equivalent. There is also a limit on the amount of data that can be retrieved at one time from eUtils. NCBI recommends that no more than 100,000 publications be retrieved from a single eUtils query ( http://www.ncbi.nlm.nih.gov/books/NBK25499). Large data sets consisting of more than 100,000 records can be retrieved incrementally (i.e. 100,000 records at a time). There is also a limit set on the frequency of eUtils requests. A maximum of three requests is allowed per second ( http://www.ncbi.nlm.nih.gov/books/NBK25497/). Violating these suggested limits may result in NCBI blocking the IP address of the offender.

Scopus and Web of Science (via InCites) are supported via file import. A user must manually export query results via the respective web interface. Scopus CSV exports are supported by the app. InCites reports must be saved in Excel 2007 (XLSX) format to be input into the app. The app can recognize InCites spreadsheets with exactly six columns in the following order (from left to right): *times cited, expected citations, publication year, subject area, all authors* and *document title*. Instructions on how to export results from InCites to this format are provided at http://baderlab.org/UserguideSocialNetworkApp#InCites.

## Results and discussion

### Use cases

We demonstrate the app using an example from the Hughes *et al.* study ^6^ in which social network analysis was used to determine whether Alzheimer Disease Centers (ADCs) based in the United States foster collaborative research. As part of the analysis, the study authors constructed multiple co-authorship networks using publication data collected from PubMed. In the original publication, the authors created Ruby scripts to query PubMed for co-authorships for a set of over 2000 researchers affiliated with ADCs.

**Figure 2.**
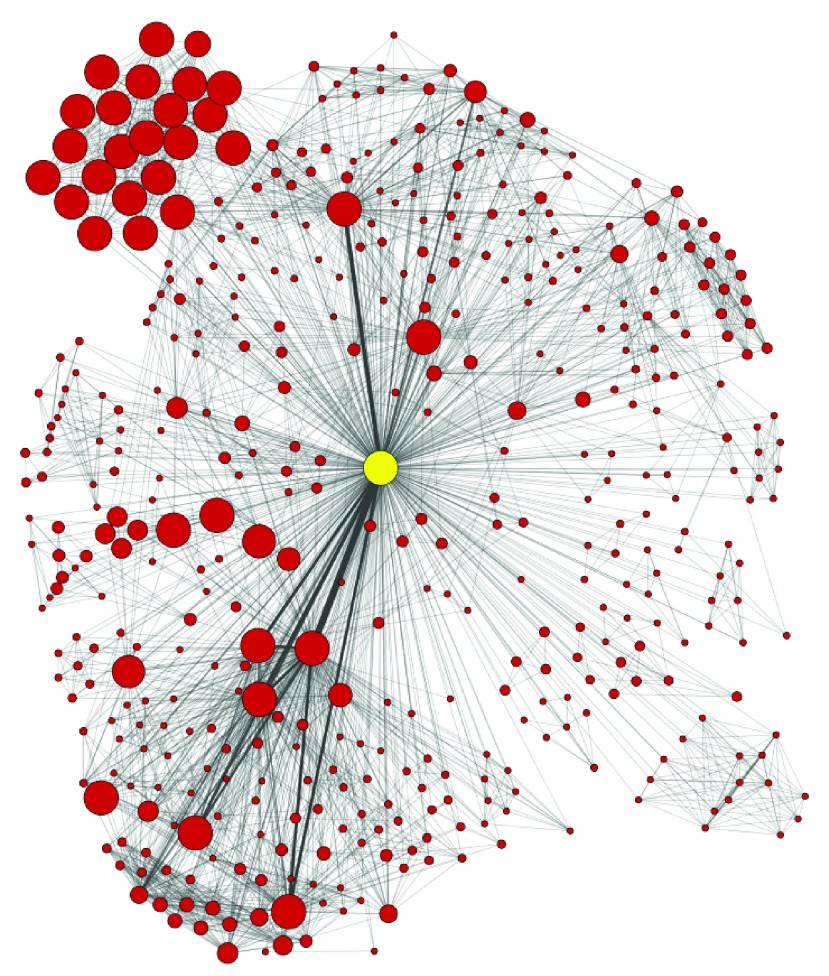
Publication network for an individual researcher at Rush University Medical Center. Each node represents a co-author of the original query author (the highlighted yellow node). The network was created by entering the author’s last name and first initial into the PubMed query bar within the Social Network App. The network was then automatically created. The yFiles Organic layout was applied to better visualize the network. Node size represents the cumulative number of the author’s publication citation counts as automatically retrieved from PubMed based on the set of publications associated with the node (the count only includes citations of publications that are in PubMed Central). Thickness of the edges connecting the nodes represents the number of publications the two authors have published together.

The simplest way to interact with the app is to create either an individual researcher’s publication network or a co-authorship network for an individual organization. Using an individual author from a single ADC, Rush University Medical Center, Figure 2 shows an individual’s publication network. We created the co-authorship network by entering the researcher’s name (last name <space> first initial, as expected by Pubmed) into the PubMed search bar (see Figure 1) and clicking on the ’Create Network’ button. In order to generate the co-authorship network the app parses the results returned from the query. We assumed that all the authors with the specified last name and first initial in the results correspond to a single author. However, we made no attempt at name disambiguation, thus authors with common last names may be associated with inflated numbers of publications. For an individual author the same process can be performed on the Scopus or Incites websites to retrieve output files that can be loaded by the app. Conflicting author names may still be present although having institution affiliations available – as is the case for Scopus and InCites exported data – can help in disambiguating authors. Until such time that databases become cleaner or reliable automatic name disambiguation services become available, we recommend that users manually clean their data to resolve errors and name ambiguities before relying on co-authorship network results to support important decisions. The main difference between networks generated by PubMed, Scopus and Incites is the number of citations attributed to each author. PubMed counts paper citations only for articles found in the freely available PubMed Central literature archive whereas Scopus and Incites use a much larger set of publications stored in their databases. Thus, Scopus and Incites provide more accurate citation counts.

**Figure 3.**
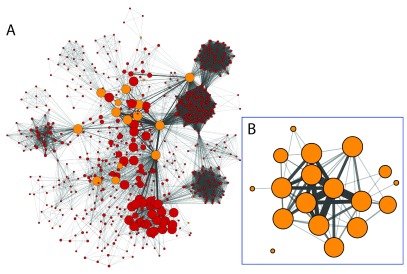
Co-authorship networks for ADC researchers at Rush University Medical Center. ( **A**) Each node represents an author from the set of publications that have at least one Rush ADC researcher. Orange nodes are Rush ADC researchers and red nodes are non-Rush ADC researchers. Rush ADC researchers were selected manually and their node fill color was modified using the style bypass option and set to orange. This can also be achieved by importing a node attribute mapping only to the query authors and using the imported attribute to select the nodes. Node size represents the cumulative number of the author’s publication citation counts as automatically retrieved from PubMed based on the set of publications associated with the node (the count only includes citations of publications that are in PubMed Central). Edge thickness represents the number of publications the two authors have published together. ( **B**) Subset of the network in ( **A**) containing only the ADC researchers. Author names are not shown to reduce visual clutter and to protect anonymity. Large cliques represent many-author publications.

Extending the simple use case, using all authors from a single institution, such as Rush University Medical Center, as a query, Figure 3A shows the resulting co-authorship network (searching PubMed for publications that have at least two Rush University ADC researchers). Figure 3B shows the co-authorship network for only the Rush University ADC researchers. The length of the query depends on the number of researchers in the query set and would have the following format:

**Example Department PubMed Query**

( ("LastName1 FirstInitial1"[Au] AND "LastName2 FirstInitial2"[Au] ) OR

("LastName1 FirstInitial1"[Au] AND "LastName3 FirstInitial3"[Au] ) OR

("LastName1 FirstInitial1"[Au] AND "LastName4 FirstInitial4"[Au] ) OR

("LastName2 FirstInitial2"[Au] AND "LastName3 FirstInitial3"[Au] ) OR

("LastName2 FirstInitial2"[Au] AND "LastName4 FirstInitial4"[Au] ) OR ... )

AND "Rush University Medical Center"

**Figure 4.**
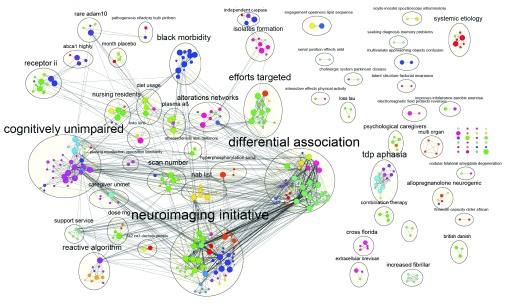
Co-authorship network for all ADC researchers from Alzheimer’s publications in 2010. Each node represents an ADC researcher and its color represents their institution (as specified in the original dataset). Node colors were automatically generated in the visual style for the ‘original institution’ attribute that was available for this author set and loaded onto the co-authorship network after its creation. Node size represents the cumulative number of the author’s publication citation counts as automatically retrieved from PubMed based on the set of publications associated with the node (the count only includes citations of publications that are in PubMed Central). Thickness of the edges connecting the nodes represents the number of publications the two authors have published together. Each cluster of authors, as calculated by the clusterMaker app ^4^ , is annotated with an additional circle around its members. Cluster labels aim to summarize the group with the two most frequent words in the author’s set of publication titles as computed by the WordCloud App ^5^).

A university department, faculty or a collaborative group typically desires to visualize and analyze all publications from the organizational unit over a period of time to help evaluate research productivity and effectiveness. Also, users may be interested in visualizing all of the publications and their topics in a particular research area. To demonstrate how the app can be used for a more sophisticated use case that also highlights how Cytoscape features can be used as part of a workflow, a simple comparison to the original broad analysis Hughes *et al.* was performed. We queried PubMed for the same set of researchers as used in the Hughes *et al.* study. Each author was queried along with their institution to reduce false positives and the entire query was limited to publications containing “alzheimer”. The set of authors was large, leading to the creation of a large PubMed query, thus the PubMed web interface was used to execute the query. Both the Scopus and InCites web interfaces were unable to process the query and it was too long to pass to eUtils. Limiting the query to papers published in 2010 returned a set of 382 publications. Using the PubMed XML file downloaded from the PubMed website, we constructed a co-authorship network. By using Cytoscape’s filtering capabilities, we reduced the network to just the authors used in the original query (see Figure 4). With Cytoscape’s Styles, we colored nodes by institution as specified in the original dataset. To summarize this network we used a feature in the Enrichment Map App ^7^ that makes use of two other Cytoscape apps (clusterMaker ^4^ and WordCloud ^5^) to automatically cluster and annotate the network based on the word summaries of a given attribute. Each cluster was annotated using frequent words found in the titles of publications within each cluster. This automatically highlights the collaborative research topics included in the network. The network can be further reduced by creating groups associated with each cluster. By collapsing the groups to an individual node the complexity in the network would be substantially reduced and the resulting network would highlight research themes found in this set of publications.

This workflow also illustrates the challenges of working with large co-authorship networks and large networks in general. There are many Cytoscape features and apps that can be used to reduce complexity of the network and help summarize the results. Given the limits of searching in PubMed, Scopus and Incites, a broad global analysis similar to the one conducted by Hughes *et al.* likely requires multiple queries, possibly automated by scripts to retrieve different data from the databases along with a process to collate and filter the results to generate the final set of publications to be analyzed. This is currently beyond the scope of this app, but more complex and automated query functionality could be added in the future.

## Conclusions

Users interested in creating visual summaries of individuals connected via co-authorship links in academia will benefit from the Social Network App. The app aids users unfamiliar with Cytoscape by providing an intuitive and navigable user interface to query multiple bibliographic databases. Advanced users who wish to analyze large networks can take advantage of many powerful Cytoscape features. In the future we plan to expand the Social Network App to enable the creation of co-authorship networks made from multiple PubMed queries or files as well as support for Cytoscape commands which would enable scripted access to the app. We also plan to implement a system that will help disambiguate authors with common names and add support to visualize connections formed by Twitter, LinkedIn and Facebook users.

## Software availability

### Software available from

http://apps.cytoscape.org/apps/socialnetworkapp

### Latest source code

https://github.com/BaderLab/SocialNetworkApp

### Source code as at the time of publication

https://github.com/F1000Research/SocialNetworkApp

### Archived source code as at time of publication

http://dx.doi.org/10.5281/zenodo.19825 ^8^

### License: Lesser GNU Public License 2.1

https://www.gnu.org/licenses/old-licenses/lgpl-2.1.html

### Tutorials

http://baderlab.org/UserguideSocialNetworkApp
