# Supplementary material for: Social Network: a Cytoscape app for visualizing co-authorship networks
Source: F1000Res. 2015 Dec 23;4:481. Originally published 2015 Aug 5. [Version 3] doi: 10.12688/f1000research.6804.3 (PMC4760270; doi:10.12688/f1000research.6804.3)
Supplement: Peer review history

# Peer review: Referee response for version 3

**DOI:** 10.5256/f1000research.8161.r12522

**Published:** 2016-02-18

**Authors:** Shahadat Uddin

**Affiliations:** 1 Faculty of Engineering & Information Technology, University of Sydney, Sydney, NSW, Australia

The authors need to clearly articulate the contribution of the paper. In its present form, it seems that it is a paper that describes an app for co-authorship analysis with some examples of this kind of analysis. If this is the main aim then the authors do not need the methods section.

I have read this submission. I believe that I have an appropriate level of expertise to confirm that it is of an acceptable scientific standard, however I have significant reservations, as outlined above.

# Peer review: Referee response for version 3

**DOI:** 10.5256/f1000research.8161.r11782

**Published:** 2016-01-04

**Authors:** Michael Bales, Terrie Wheeler

**Affiliations:** 1 Weill Cornell Medical College, New York, NY, USA

The authors have fully addressed our comments.

We have read this submission. We believe that we have an appropriate level of expertise to confirm that it is of an acceptable scientific standard.

# Peer review: Referee response for version 2

**DOI:** 10.5256/f1000research.7716.r10730

**Published:** 2015-10-15

**Authors:** Michael Bales, Terrie Wheeler

**Affiliations:** 1 Weill Cornell Medical College, New York, NY, USA

Thank you for responding to our comments. We just have one concern remaining, in reference to the following discussion:

In our initial review we wrote, "On a related note, later in the sixth paragraph of the 'Database evaluation and implementation' section, the authors state that 'There is also a limit on the amount of data that can be retrieved at one time from eUtils. NCBI recommends that no more than 500 publications be retrieved from a single eUtils query'. The citation given is http://www.ncbi.nlm.nih.gov/books/NBK25498/. However, this citation does not directly support this assertion.****

You responded: "Our apologies for the wrong citation. In “Building Customized Data Pipelines Using The Entrez Programming Utilities” ( http://www.ncbi.nlm.nih.gov/books/NBK1058/) under the “Handling Large Datasets” subheading, it is stated that large lists should be split into smaller batches of around 500 records. We have updated the manuscript to reflect this change."

However, the new citation you provided refers to uploading UIDs, not downloading retrieved data. While it may be necessary when using Scopus, it is not necessary when using eUtils to modify queries so as to retrieve records in small batches. The actual maximum number of records that can be retrieved by a single query is 100,000, as indicated at http://www.ncbi.nlm.nih.gov/books/NBK25499/: "Increasing retmax allows more of the retrieved UIDs to be included in the XML output, up to a maximum of 100,000 records." One of us (M.B.) has used eUtils within the last year to download many thousands of records at a time, and can personally confirm that this is indeed possible.

We have read this submission. We believe that we have an appropriate level of expertise to confirm that it is of an acceptable scientific standard, however we have significant reservations, as outlined above.

# Response

**Published:** 2015-12-08

**Authors:** Victor Kofia

**Affiliations:** University of Toronto, Canada

Thanks for pointing out this documentation page. Our app uses esearch for UID retrieval and esummary for document summaries which we use to gather detailed information for the app. These have retmax limits of 100,000 and 10,000, respectively and we also confirmed these. We have switched the reference link to http://www.ncbi.nlm.nih.gov/books/NBK25499/ and updated our manuscript in this regard.

# Peer review: Referee response for version 1

**DOI:** 10.5256/f1000research.7315.r9866

**Published:** 2015-08-21

**Authors:** Jiang Bian

**Affiliations:** 1 Department of Health Outcomes & Policy, University of Florida, Gainesville, FL, USA

The article describes a software as an extension of the Cytoscape that can automatically query popular citation databases (PubMed only) and derive co-authorship networks based on the query results. It's a laudable goal, and will be a welcomed tool for researches on collaboration networks. Especially, the tool is disseminated as a open-source tool. However, there are a number of concerns.

1. The software does not have any process for disambiguation. This is problematic. In general, citation databases do not provide disambiguation services. Google Scholar attempts to "learn" which publications belong to a specific author when creating the author profile. However, it is not very accurate either at the beginning for common names. For studying social networks, getting accurate information is important, especially for studying Ego networks that focus on a specific person.
2. It is not very user friendly in terms of gathering data. Only for Pubmed, users can enter queries through the tool. With other citation databases (InCites and Scopus), users will need to query the databases directly and then export the results. It is understandable that this is due to InCites and Scopus only provide paid API services. However, it is unclear whether the authors have implemented such integration for users who have paid those API services.
3. Further, even for Pubmed search, the software relies on the users to understand Pubmed query syntax. It would be useful if the authors could provide (in addition to text Pubmed query) guided search (see Pubmed Advanced search) for common use cases in studying collaboration networks. It would be very useful, if the authors could allow users to use the same guided search interface to three all citation databases.

I have read this submission. I believe that I have an appropriate level of expertise to confirm that it is of an acceptable scientific standard, however I have significant reservations, as outlined above.

# Response

**Published:** 2015-09-27

**Authors:** Victor Kofia

**Affiliations:** University of Toronto, Canada

**The software does not have any process for disambiguation. This is problematic. In general, citation databases do not provide disambiguation services. Google Scholar attempts to "learn" which publications belong to a specific author when creating the author profile. However, it is not very accurate either at the beginning for common names. For studying social networks, getting accurate information is important, especially for studying Ego networks that focus on a specific person.**

Thank you for the comment.  We agree that name disambiguation is a key concern with co-authorship networks.  This is something we hope to address in the app in the future.  In the meantime we have added to the paper some tips to minimize this issue. As noted in response to referee 1, until such time that databases become cleaner or reliable automatic name disambiguation services become available, we recommend that users manually clean their data to resolve errors and name ambiguities before relying on co-authorship network results to support important decisions.

**It is not very user friendly in terms of gathering data. Only for Pubmed, users can enter queries through the tool. With other citation databases (InCites and Scopus), users will need to query the databases directly and then export the results. It is understandable that this is due to InCites and Scopus only provide paid API services. However, it is unclear whether the authors have implemented such integration for users who have paid those API services.**

Our apologies for the lack of clarity. Right now the app does not support API querying for users who have paid services, but we have added this to a list of planned features in our github issue tracker at https://github.com/BaderLab/SocialNetworkApp/issues

**Further, even for Pubmed search, the software relies on the users to understand Pubmed query syntax. It would be useful if the authors could provide (in addition to text Pubmed query) guided search (see Pubmed Advanced search) for common use cases in studying collaboration networks. It would be very useful, if the authors could allow users to use the same guided search interface to three all citation databases.**

Thanks for the comment. We have improved the user interface of the app and included a link to the PubMed query syntax. We have added a feature request to our issue tracker to develop a user friendly interface to assist users who may not be familiar with PubMed query syntax. For now we have included in the manuscript links to the relevant PubMed tutorials.

# Peer review: Referee response for version 1

**DOI:** 10.5256/f1000research.7315.r9859

**Published:** 2015-08-14

**Authors:** Michael Bales, Terrie Wheeler

**Affiliations:** 1 Weill Cornell Medical College, New York, NY, USA

The authors describe a tool that allows users to create co-authorship network diagrams within the Cytoscape application. This tool simplifies the network production process into as few as two steps: users can enter a PubMed query and then click the “search” button. A co-authorship network is generated automatically and displayed in Cytoscape; users can then use features available within Cytoscape to carry out additional tasks, such as adjusting visual properties and measuring topological measures of network structure. The system also supports searches to Scopus and Web of Science (via InCites).

The authors illustrate the system’s design and functions, describe how to use it, and present a use case in which they use the tool to replicate a search carried out in a study by Hughes *et al*. In so doing they highlight several challenges that may occur when working with large co-authorship networks.

The authors contend that co-authorship network visualization can be useful for understanding collaborative research networks or for “communicating the extent of collaboration and publication productivity among a group of researchers”. In this paper the authors do not conceive of a method to evaluate their app, for example to assess satisfaction with the app among system users, or to see whether system users are able to integrate the app into meaningful workflows. The authors report that they plan to continue developing the app, for example, by extending it so that it can display data from Facebook, Twitter, and LinkedIn.

The availability of the software is a welcome addition to existing tools for co-authorship network production. Its automated features and relatively seamless integration into Cytoscape will (we expect) make it an appealing option for analysts; to the extent possible it takes care of tedious steps that analysts may be accustomed to carrying out manually.

We have identified several minor points for the authors to take into consideration.

First, in the sixth paragraph of the “Database evaluation and implementation” section, the authors mention that E-utilities queries are limited to several hundred characters. While this may be the case for standard E-utilities queries, it is possible by using an HTTP Post call to make significantly longer queries. From Sayers E. The E-Utilities In-Depth: Parameters, Syntax, and More, http://www.ncbi.nlm.nih.gov/books/NBK25499/: “For very long queries (more than several hundred characters long), consider using an HTTP POST call.”

In the version of the Social Network that was current as of the time of this review, one of us (M.B.) attempted a query containing more than 1,000 characters and it was successful. In any case we would like to request clarification, as it appears that the current version of the system may be capable of longer queries, possibly by doing an HTTP Post request.

On a related note, later in the sixth paragraph of the “Database evaluation and implementation” section, the authors state that “There is also a limit on the amount of data that can be retrieved at one time from eUtils. NCBI recommends that no more than 500 publications be retrieved from a single eUtils query”. The citation given is http://www.ncbi.nlm.nih.gov/books/NBK25498/. However, this citation does not directly support this assertion. It is true, as the authors also point out, that requests should be limited to a maximum of three per second. Additionally, large jobs are to be limited to nights and weekends Eastern time ( http://www.ncbi.nlm.nih.gov/books/NBK25497/). However, if there is a stated recommendation that no more than 500 publications be retrieved from a single eUtils query, we request that the authors identify a different source in which this is indicated in writing.

In the fourth paragraph of the “Database evaluation and implementation” section the authors mention that “Scopus and Web of Science APIs require paid subscriptions”. It is our understanding that Web of Science has an API that is free to subscribers, in addition to a paid API with more data fields. We would like clarification on whether the authors were referring to the API that is free to subscribers.

In the second paragraph of the “Results and discussion” section the authors state, “We created the co-publication network by entering the researcher’s name (last name <space> first initial, as expected by PubMed) … and clicking on the search button”. Due to the problem of ambiguous names in PubMed, it should be noted that this approach, without an attempt at name disambiguation, may result in many false drops, leading to invalid networks.

In the third paragraph of the “Methods and implementation” section the authors mention that the user panel includes a co-publication network summary panel. Later in this paragraph they mention “charts that summarize the total number of publications and citations by location can be viewed by clicking on links in the panel that navigate to summary charts created with the Google Chart API.” We have thus far been unable to locate the network summary panel or the links to the charts, so further detail would be helpful here to describe the circumstances under which these features may be used, and/or how to activate and find them.

We also have some minor editorial suggestions.

First, we wanted to point out that “co-authorship” networks is far more common in the literature than “co-publication” networks, so the authors may wish to switch to this term if desired.

Second, in the third paragraph of the “Methods and Implementation” section the authors point out that “Because InCites networks contain institutional affiliations for all the authors of a given publication, they have richer summaries.” It may also be worth pointing out here that Scopus does this as well.

Third, the sixth paragraph of the “Database evaluation and implementation” section contains a broken link ( http://baderlab.org/Software/SocialNetworkApp#PubMed).

We applaud the authors for making this app available for use within a freely available tool that has an active user base and community of users, and are hopeful that the authors will continue with active development of this tool, so that they may be responsive to user suggestions that may further improve the user experience and integration into workflows.

We have read this submission. We believe that we have an appropriate level of expertise to confirm that it is of an acceptable scientific standard, however we have significant reservations, as outlined above.

# Response

**Published:** 2015-09-27

**Authors:** Victor Kofia

**Affiliations:** University of Toronto, Canada

**In the version of the Social Network that was current as of the time of this review, one of us (M.B.) attempted a query containing more than 1,000 characters and it was successful. In any case we would like to request clarification, as it appears that the current version of the system may be capable of longer queries, possibly by doing an HTTP Post request.**

Thanks for pointing this out. We have now modified the app to use POST for all queries, which will be part of the next release.

**On a related note, later in the sixth paragraph of the “Database evaluation and implementation” section, the authors state that “There is also a limit on the amount of data that can be retrieved at one time from eUtils. NCBI recommends that no more than 500 publications be retrieved from a single eUtils query”. The citation given is http://www.ncbi.nlm.nih.gov/books/NBK25498/. However, this citation does not directly support this assertion.**

Our apologies for the wrong citation. In “Building Customized Data Pipelines Using The Entrez Programming Utilities” ( http://www.ncbi.nlm.nih.gov/books/NBK1058/) under the “Handling Large Datasets” subheading, it is stated that large lists should be split into smaller batches of around 500 records. We have updated the manuscript to reflect this change.

**In the fourth paragraph of the “Database evaluation and implementation” section the authors mention that “Scopus and Web of Science APIs require paid subscriptions”. It is our understanding that Web of Science has an API that is free to subscribers, in addition to a paid API with more data fields. We would like clarification on whether the authors were referring to the API that is free to subscribers.**

We were referring to both APIs. Since users have to be subscribed to Web of Science to access the ‘free’ API we classified it as ‘requiring a paid subscription’.  We have clarified this point in the revision.

**In the second paragraph of the “Results and discussion” section the authors state, “We created the co-publication network by entering the researcher’s name (last name <space> first initial, as expected by PubMed) … and clicking on the search button”. Due to the problem of ambiguous names in PubMed, it should be noted that this approach, without an attempt at name disambiguation, may result in many false drops, leading to invalid networks.**

We agree with the reviewer. Name disambiguation is definitely a problem when constructing co-authorship networks in this way. And it is a problem that affects all databases. We have updated the text to reflect this as it is something that all readers should be aware of. Until such time that databases become cleaner or reliable automatic name disambiguation services become available, we recommend that users manually clean their data to resolve errors and name ambiguities before relying on co-authorship network results to support important decisions.

**In the third paragraph of the “Methods and implementation” section the authors mention that the user panel includes a co-publication network summary panel. Later in this paragraph they mention “charts that summarize the total number of publications and citations by location can be viewed by clicking on links in the panel that navigate to summary charts created with the Google Chart API.” We have thus far been unable to locate the network summary panel or the links to the charts, so further detail would be helpful here to describe the circumstances under which these features may be used, and/or how to activate and find them.**

The network summary panel is located at the bottom of the user panel. Instead of a figure that only shows the top half of the panel we have included a new figure that contains the entire panel as this will enable readers to view the sections we are referring to. If no network summary panel exists in your setup and the problem persists then it may be a bug, in which case we would appreciate it being filed as a bug report on GitHub (https://github.com/BaderLab/SocialNetworkApp/issues) or emailed to us so we can fix it.

Also note that even with the network summary panel being visible, links to the charts will only appear after an InCites network has been created. No charts are available for Scopus and PubMed networks at the moment. So please verify that you are using an InCites document to build the network. An example InCites document is provided in the user guide: http://baderlab.org/Software/SocialNetworkApp. We have made this clearer in the text.

**We also have some minor editorial suggestions. First, we wanted to point out that “co-authorship” networks is far more common in the literature than “co-publication” networks, so the authors may wish to switch to this term if desired.**

Thank you for pointing this out. We have switched to using the more common “co-authorship” term throughout the manuscript.

**Second, in the third paragraph of the “Methods and Implementation” section the authors point out that “Because InCites networks contain institutional affiliations for all the authors of a given publication, they have richer summaries.” It may also be worth pointing out here that Scopus does this as well.**

It is indeed true that Scopus provides institutional affiliations. The default setting for exporting files from Scopus is “Citation Information Only” which does not include the institutional affiliations of the co-authors. We have extended the functionality so that the app is capable of parsing Scopus reports that contain additional information (like institutional affiliations). We have made a note of this in the online manual.

**Third, the sixth paragraph of the “Database evaluation and implementation” section contains a broken link ( http://baderlab.org/Software/SocialNetworkApp#PubMed).**

Thanks for noticing this. We have updated the manuscript to include the correct link: http://baderlab.org/UserguideSocialNetworkApp#PubMed

**We applaud the authors for making this app available for use within a freely available tool that has an active user base and community of users, and are hopeful that the authors will continue with active development of this tool, so that they may be responsive to user suggestions that may further improve the user experience and integration into workflows.**

Thank you for taking the time to review our app.
